# Features of Psychotic Symptoms in Methamphetamine Use Disorder Patients and Ketamine Use Disorder Patients: A Cross-Sectional Study

**DOI:** 10.3389/fpsyt.2021.786622

**Published:** 2022-01-18

**Authors:** Tao Luo, Meng Xiao, Chang Qi, Qiuxia Wu, Jinsong Tang, Yanhui Liao

**Affiliations:** ^1^Department of Psychology, The First Affiliated Hospital of Nanchang University, Nanchang, China; ^2^The Fourth People's Hospital of Urumqi, Urumqi, China; ^3^Department of Psychiatry, Zhejiang Provincial People's Hospital, People's Hospital of Hangzhou Medical College, Hangzhou, China; ^4^Department of Psychiatry, The Second Xiangya Hospital, Central South University, Changsha, China; ^5^Department of Psychiatry, Sir Run Run Shaw Hospital, Zhejiang University School of Medicine, Hangzhou, China

**Keywords:** methamphetamine use disorder, ketamine use disorder, psychotic symptoms, positive symptoms, negative symptoms, schizophrenia

## Abstract

**Background:**

Methamphetamine and ketamine are commonly used club drugs. Both of them have been reported to mimic psychotic symptoms of schizophrenia. However, the prevalence and detailed features of psychotic symptoms among methamphetamine use disorder (MUD) and ketamine use disorder (KUD) patients are largely unknown. This study aimed to measure psychotic symptoms among patients with MUD and KUD.

**Methods:**

A total sample of 842 patients from voluntary drug rehabilitation centers, including 462 MUD patients and 380 KUD patients, were invited to this study. The Positive and Negative Syndrome Scale (PANSS) was applied to assess psychotic symptoms in these two groups of patients.

**Results:**

The prevalence of psychotic symptoms was significantly higher among MUD patients than KUD patients (75.1 vs. 50.5%, 95% CI: 3.532 – 11.858, *p* < 0.001). Compared with KUD patients, MUD patients were more likely to experience positive symptoms (PANSS positive scores: 11.5 ± 6.07 vs. 15.1 ± 8.22, *P* < 0.001) and negative symptoms (PANSS negative scores: 12.4 ± 6.60 vs. 14.5 ± 8.63, *P* < 0.001), but not general symptoms (PANSS general scores: 31.2 ± 13.90 vs. 32.2 ± 15.13, *P* < 0.001).

**Conclusions:**

The current study found that more than half of MUD and KUD patients experienced psychotic symptoms, and that patients with MUD are more likely to experience positive and negative symptoms than patients with KUD. The findings provide a new perspective for exploring the neuropathological mechanism of psychotic symptoms of schizophrenia.

## Introduction

The use of synthetic drugs such as methamphetamine and ketamine has been recognized as an increasingly serious and important public health issue in China and other countries (1–3). A series of studies indicate that chronic use of both methamphetamine and ketamine can lead to psychotic symptoms (4–6), which are mainly manifested as persistent hallucinations, delusions, and negative symptoms.

The experience of psychotic symptoms ranged from 26 to 46% among people with methamphetamine use disorder (MUD) (7). A study with 292 MUD patients from a drug rehabilitation center in Malaysia showed that 47.9% of them had a history of psychotic symptoms (8). Studies show that patients with ketamine use disorder (KUD) have a relatively lower risk of psychotic symptoms than those of MUD patients, around 8% (9). In drug-free healthy individuals, low-dose administration of either ketamine or amphetamine produced positive symptoms (such as conceptual disorganization) and euphoria. Ketamine mainly produced perceptual changes, concrete ideation, and unusual mannerisms, negative symptoms, and disrupted delayed recall. Amphetamine mainly produced hostility, grandiosity, and somatic concern. The findings from the above study indicate that glutamate and DA may differentially contribute to psychosis (10). A recent systematic review and meta-analysis further confirmed that ketamine induced psychosis-like symptoms in healthy volunteers (11).

The neuropathological mechanisms of psychotic symptoms are not fully understood. Both methamphetamine and ketamine use-induced psychotic symptoms can mimic symptoms of schizophrenia. Methamphetamine has been used for the study of the dopamine (DA) model of schizophrenia (12). Ketamine, as an N-methyl-D-aspartate (NMDA) antagonist, has often been studied for the glutamate model of schizophrenia (13). DA plays a well-recognized role in a variety of neurophysiologic functions such as cognition, mood and reward (14). Methamphetamine dysregulates both DA transmission and DA reuptake, and it mainly acts on DA transporter (DAT) and the vesicular monoamine transporter 2 (VMAT2) to inhibit the reabsorption of DA and promote the release of DA in the Nucleus Accumbens (NA) *via* the sigma receptor (15). Ketamine primarily impairs NMDA glutamate receptor neurotransmission, and it has been suggested to increase cortical glutamate release via indirect inhibition of GABAergic interneurons, implicating in the pathophysiology of schizophrenia (16).

Although a line of study has shown that the psychotic symptoms caused by methamphetamine and ketamine are similar to those of schizophrenia. The prevalence and detailed features of psychotic symptoms (including positive, negative and general symptoms) among methamphetamine use disorder (MUD) and ketamine use disorder (KUD) patients are largely unknown. The aim of this study was to measure psychotic symptoms among patients with MUD and KUD. Based on previous studies, this study assumed that the prevalence of positive and negative symptoms would be higher among MUD patients than KUD patients. The findings from this study may provide a preliminary basis for clinical interventions of MUD and KUD associated psychosis, and give a new perspective for exploring the neuropathological mechanism of psychotic symptoms of schizophrenia.

## Methods

### Recruitment

This cross-sectional study assessed the prevalence of psychotic symptoms in a clinic-based sample of KUD patients and MUD patients. We recruited 380 KUD patients and 462 MUD patients from two rehabilitation centers in mainland China (Guangzhou Baiyun Voluntary Drug Rehabilitation Center in Guangdong Province and Kangda Voluntary Drug Rehabilitation Center in Hunan Province) from January 2012 to October 2016. Drug use (methamphetamine or ketamine) was validated via urine toxicology screen and self-report data. All participants were diagnosed through a semi-structured interview (Structured Clinical Interview for DSM-IV-TR, Axis I, Patient Version, SCID-I/P) by experienced psychiatrists (with more than 5 years of clinical experience). The Positive and Negative Syndrome Scale (PANSS) was used to assess psychotic symptoms in these two groups of patients. Before enrolling patients into this study, all psychiatrists who involved in current study were trained to administer the PANSS and SCID-I/P with good reliability (>0.80). The inclusion criteria were: inpatients from drug rehabilitation center; after detoxification and had 2 weeks of drug abstinence; met diagnosis for MUD or KUD; used methamphetamine or ketamine for more than 12 months; 18 years of age or older; willing to participant, being able to communicate in Chinese. The exclusion criteria were: suffering from other severe mental and/or physical illnesses; have a history of brain trauma; and have other substance use disorders (except for nicotine).

### Ethics

Informed consent was obtained from all patients in the study. The procedures were performed in accordance with the Declaration of Helsinki. The ethical approval for this study was obtained from the Ethics Committee of the Second Xiangya Hospital, Central South University (No. S163, 2011).

### Measures

#### Sociodemographic and Drug Use Information

Sociodemographic and drug use information, such as age, gender, education, age of the first use, quantity of drug (g) per time, drug craving, and times of rehabilitation were collected by self-reported questionnaires. Methamphetamine or ketamine craving was assessed by the Visual Analog Scale for Craving (VASc) (17). The VASc displays a scale from 0 (left, no craving) to 10 (right, most extreme craving).

#### The Positive and Negative Syndrome Scale

The Positive and Negative Syndrome Scale (18) is a 30-item scale designed to obtain a measure of positive (items P1 to P7) and negative (items N1 to N7) symptoms in schizophrenic patients, as well as a measure of general psychopathology (items G1 to G16). All 30 items are rated on a 7-point scale: 1 = absent, 2 = minimal, 3 = mild, 4 = moderate, 5 = moderate–severe, 6 = severe, 7 = extreme. Factorial analyses of subscales found that a five-factor model better captures PANSS structure in schizophrenia samples (19). In the five-factor model, smaller groupings of items represent the following symptoms: positive (items P1, P3, P5, G9), negative (items N1, N2, N3, N4, N6, G7), disorganized/concrete (items P2, N5, G11), excited (items P4, P7, G8, G14), and depressed (items G2, G3, G6) symptoms.

### Statistical Analyses

Statistical analysis was performed using the SPSS for Windows (Version 23, SPSS Inc., Chicago, IL, USA) software package. Descriptive statistics were used to examine demographic, drug use, and psychotic characteristics. Independent sample *t*-tests or χ^2^-square tests were performed to determine group differences in demographic characteristics, substance use profiles, and psychotic symptoms between these two groups. Multiple linear regression models have been applied to evaluate the impact of types of used drugs (methamphetamine or ketamine), gender, age, age of first-time drug use, duration of drug use (months), and frequency during the last drug use month on psychotic symptoms (PANSS total score). An alpha level of 0.01 was set to determine statistical significance.

## Results

### Sociodemographic and Drug Use Characteristics

This study included 842 drug users (462 MUD patients and 380 KUD patients). Demographic and drug use characteristics of patients with MUD and KUD are shown in Table 1.

**Table 1:** Sample characteristics.

	**MUD patients (*n* = 462)**	**KUD patients (*n* = 380)**	**t/χ^2^**	***p***
**Demographic variables**				
Age, years	29.4 ± 6.32	26.8 ± 5.36	6.39	<0.001
Range, years	14–53	16–52		
Female	28 (6.1%)	43 (11.3%)	−7.46	0.006
Education level, years,	11.2 ± 2.78	11.6 ± 2.50	−2.58	0.010
Unmarried	224 (48.5%)	224 (58.9%)	−9.17	0.002
Unemployed	98 (21.2%)	78 (20.5%)	0.06	0.808
Body mass index	23.3 ± 3.56	21.8 ± 4.20	5.68	<0.001
**Drug use variables**				
Age of the first use	25.5 ± 6.45	21.9 ± 5.29	8.88	<0.001
Duration, months,	43.1 ± 30.12	57.5 ± 32.31	−6.63	<0.001
Range, months,	2–204	1–157		
Times of drug use per day during the last 12 months	2.4 ± 1.08	1.8 ± 1.06	7.54	<0.001
Times of drug use per day during the last 3 months	2.4 ± 1.20	1.7 ± 1.07	9.03	<0.001
Quantity of drug (g) per time	0.6 ± 0.79	1.2 ± 1.28	−9.13	<0.001
Craving by VASc	3.5 ± 2.78	5.3 ± 2.80	−9.01	<0.001
Times of rehabilitation	1.4 ± 1.65	1.6 ± 2.34	−1.88	0.060
**Cigarette smoking**	444 (96.1%)	367 (96.6%)	−0.11	0.716
Cigarettes per day	19.8 ± 10.72	18.7 ± 8.17	1.68	0.094
Duration, years	11.7 ± 5.68	9.4 ± 5.10	5.59	<0.001
**Alcohol drinking**	137 (29.7%)	133 (35.0%)	−2.74	0.098
Duration (years)	10.0 ± 6.12	8.2 ± 5.14	2.39	0.018
**Other drugs use** ^**a**^				
Methamphetamine	—	123 (32.4%)		
Ketamine	55 (11.9%)	—		
Ma Gu (amphetamine+caffeine)	166 (35.9%)	70 (18.4%)		
Ecstasy	40 (8.7%)	61 (16.1%)		
Marijuana	11 (2.4%)	20 (5.3%)		
“Happy Water”^b^	5 (1.1%)	21 (5.5%)		
Heroin	17 (3.7%)	3 (0.8%)		
Diazepam	0 (0.0%)	7 (1.8%)		
Dolantin	0 (0.0%)	1 (0.3%)		
Tramadol	2 (0.4%)	0 (0.0%)		
**Psychotic symptoms**	347 (75.1%)	192 (50.5%)	54.69	<0.001
Onset age	28.5 ± 6.32	25.4 ± 5.49	6.05	<0.001
Duration (month)	15.1 ± 19.43	3.5 ± 24.53	4.01	<0.001
**PANSS total score**	61.8 ± 29.43	55.3 ± 24.57	3.48	0.001

*Data are in mean ± SD or n (%)*.

*MUD, methamphetamine use disorder; KUD, ketamine use disorder; VASc, the visual analog scale for craving*.

Significantly different from control group, p < 0.01.

a *MUD patients only had methamphetamine dependence, KUD patients only had ketamine dependence (except for nicotine). However, each person could have tried other drugs for one or more than one times*.

b *“Happy Water (Kai Xin Shui)” is a kind of mixed liquid containing club drugs such as methamphetamine, amphetamine, ketamine, ecstasy*.

### Factors Predicting Psychotic Symptoms

Multiple linear regression models were applied to evaluate the predictors (demographic and drug use factors) for psychotic symptoms (PANSS total score). As shown in Table 2, only types of used drugs (methamphetamine or ketamine) were associated with PANSS total score, indicating that MUD patients experienced more psychotic symptoms than KUD patients did (*p* < 0.001).

**Table 2:** MLR model predicting increase in PANSS total score.

**Variable**	**B**	**Beta**	***t***	**95% Confidence interval**	***p***
Types of used drugs (methamphetamine or ketamine)	7.695	0.140	3.628	3.532 to 11.858	<0.001
Gender	6.475	0.066	1.883	−0.275 to 13.224	0.060
Age	0.603	0.133	1.139	−0.436 to 1.641	0.255
Age of first-time drug use	−0.720	−0.163	−1.391	−1.736 to 0.296	0.165
Duration of drug use (M)	0.010	0.012	0.201	−0.089 to 0.110	0.841
Frequency during the last drug use month	0.470	0.020	0.564	−1.166 to 2.106	0.573

*Multiple linear regression model (MLR) model*.

*Y = 38.422 + (7.695) types of used drugs + (6.475) gender + (0.603) age – (0.720) age of first-time drug use + (0.010) duration of drug use (Months) + (0.470) frequency during the last drug use month*.

### Psychotic Symptoms in MUD Patients and KUD Patients

A total of 75.1% (*n* = 347) MUD patients and 50.5% (*n* = 192) KUD patients experienced psychotic symptoms (Table 1). Compared with KUD patients, MUD patients were more likely to suffer from psychotic symptoms (95% CI: 3.532–11.858, *p* < 0.001). The scores of PANSS were compared between these two groups. We find that compared with KUD patients, MUD patients were more likely to experience positive symptoms (11.5 ± 6.07 vs. 15.1 ± 8.22, *P* < 0.001) and negative symptoms (12.4 ± 6.60 vs. 14.5 ± 8.63, *P* < 0.001), but not general symptoms (31.2 ± 13.90 vs. 32.2 ± 15.13, *P* = 0.331) (Table 3). The proportion of each symptom in both groups, including each item of the PANSS positive, negative, and general symptoms, are also shown in Table 3. A five-factor model of positive, negative, disorganized/concrete, excited, and depressed symptoms was also compared between two groups (Table 4).

**Table 3:** Positive, negative, and general symptoms assessed by PANSS between two groups.

	**MUD patients (*n* = 462)**	**KUD patients (*n* = 380)**	**t/x^**2**^**	***p***
**Positive**	15.1 ± 8.22	11.5 ± 6.07	7.11	<0.001
p1 (Delusions)	281 (47.2%)	102 (26.8%)		
p2 (Conceptual)	223 (48.3%)	130 (34.2%)		
p3 (Hallucinations disorganization)	162 (35.1%)	122 (32.1%)		
p4 (Excitement)	256 (55.4%)	194 (51.1%)		
p5 (Grandiosity)	187 (40.5%)	110 (28.9%)		
p6 (Suspiciousness/Persecute)	242 (52.4%)	107 (28.2%)		
p7 (Hostility ion)	230 (49.8%)	114 (30.0%)		
**Negative**	14.5 ± 8.63	12.4 ± 6.60	3.78	<0.001
N1 (Blunted affect)	230 (49.8%)	192 (50.5%)		
N2 (Emotional withdrawal)	241 (52.2%)	191 (50.3%)		
N3 (Poor rapport)	226 (48.9%)	164 (43.2%)		
N4 (Passive/Apathetic social)	242 (52.4%)	200 (52.6%)		
N5 (Difficulty in abstraction withdrawal)	187 (40.5%)	115 (30.3%)		
N6 (Lack of spontaneity)	197 (42.6%)	151 (39.7%)		
N7 (Stereotyped thinking)	163 (35.3%)	106 (27.9%)		
**General**	32.2 ± 15.13	31.2 ± 13.90	0.97	0.331
G1 (Somatic concern)	253 (54.8%)	271 (71.3%)		
G2 (Anxiety)	304 (65.8%)	300 (78.9%)		
G3 (Guilt feelings)	256 (55.4%)	244 (64.2%)		
G4 (Tension)	257 (55.6%)	200 (52.6%)		
G5 (Mannerisms)	154 (33.3%)	94 (24.7%)		
G6 (Depression posturing)	235 (50.9%)	209 (55.0%)		
G7 (Motor retardation)	167 (36.1%)	145 (38.2%)		
G8 (Uncooperativeness)	161 (34.8%)	97 (25.5%)		
G9 (Unusual thought)	199 (43.1%)	105 (27.6%)		
G10 (Disorientation content)	37 (8.0%)	72 (18.9%)		
G11 (Poor attention)	203 (43.9%)	171 (45.0%)		
G12 (Lack of judgment and symptom)	240 (51.9%)	137 (36.1%)		
G13 (Disturbance of volition insight)	218 (47.2%)	210 (55.3%)		
G14 (Poor impulse control)	257 (55.6%)	208 (54.7%)		
G15 (Preoccupation)	184 (39.8%)	88 (23.2%)		
G16 (Active social avoidance)	225 (48.7%)	187 (49.2%)		

*Data are in mean ± SD or n (%)*.

*PANSS, the positive and negative syndrome scale*.

*Percentage of each symptom assessed by PANSS with ≥ 2 points (from minimal to extreme)*.

**Table 4:** PANSS scores by the five-factor model between two groups.

**PANSS scores and items**	**MUD patients**	**KUD patients**	***t***	***p***
	**Mean ±SD**	**Mean ±SD**		
**The five-factor model**				
A. Positive factor	8.2 ± 4.83	6.4 ± 3.73	6.01	<0.001
B. Negative factor	12.7 ± 7.64	11.2 ± 6.06	3.04	0.002
C. Disorganized/concrete (cognitive) factor	5.7 ± 3.09	5.1 ± 2.62	3.06	0.002
D. Excited factor	8.35 ± 4.27	7.2 ± 3.57	4.37	<0.001
E. Depressed factor	7.1 ± 3.83	7.4 ± 3.83	−1.08	0.282

*PANSS, the positive and negative syndrome scale; MUD, methamphetamine use disorder; KUD, ketamine use disorder*.

## Discussion

### Principal Findings

The current study assessed the prevalence and detailed features of psychotic symptoms among MUD and KUD patients. This study found that 75.1% MUD patients and 50.5% KUD patients from drug rehabilitation centers in China experienced psychotic symptoms, indicating that MUD patients are more likely to suffer from psychotic symptoms (including positive and negative symptoms) than KUD patients. However, MUD patients and KUD patients showed no group differences in overall general symptoms.

### Psychotic Symptoms in MUD Patients and KUD Patients

Compared with previous studies, our study found relatively high prevalence of psychotic symptoms in MUD patients and KUD patients, especially in MUD patients. One study reported that the prevalence of psychotic symptoms ranged from 26 to 46% in MUD patients (7). Another study reported that 47.9% MUD patients had history of psychotic symptoms (8). A study found that only 8% KUD had psychotic symptoms (9). However, our study found more than half of KUD patients and two-thirds of MUD patients experienced psychotic symptoms.

This study found that, compared with KUD patients, MUA patients were more likely to experience both positive and negative symptoms, but not general symptoms. Previous studies suggest that methamphetamine mainly causes psychotic symptoms by causing DA system dysfunction, acting on DA transmission in the central nervous system via the inhibition of the DA transporter and the VMAT2, and leading to the increase of DA concentration in the mesolimbic, nigrostriatum, and mesocortical, resulting in psychotic symptoms, mainly positive symptoms (6, 20). Furthermore, in the nigrostriatum, dysregulated DA neuron firing can abnormally highlight irrelevant stimuli, thereby producing percepts and thoughts with aberrant salience, resulting to delusions and hallucinations (10, 21). In terms of methamphetamine use induced negative symptoms, one possible explanation is that the reduction of the signal-to-noise ratio of adaptive phasic signaling has the potentiality to reduce the appetitive properties of a given reward, thereby leading to motivation and anhedonia, and other negative symptoms.

Research indicates that ketamine produces psychotic symptoms through dysfunction in the glutamate system. As a high affinity non-competitive antagonist of NMDAR, ketamine has been shown to mimic more negative symptoms than active symptoms of psychotic behavior in previous studies (10, 21). A line of study found that ketamine can produce positive and negative symptoms similar to schizophrenia with long-term use (22, 23), which is consistent with our results. A preclinical study found that ketamine causes psychotic episodes by significantly increasing synaptic glutamate release in the cortex and striatum (24).

High prevalence of psychosis among MUD patients and KUD patients in current study indicates that both the glutamate and DA systems directly or indirectly interact with psychotic symptoms (4, 10, 13). The psychotic symptoms were more easily induced by MA than ketamine, indicating that the dysfunction of the glutamate system may cause psychotic symptoms through indirect (not direct) action on the DA system. Methamphetamine or amphetamine induced hyper-dopaminergic states would be associated with the more vulnerability of psychosis (25). Previous studies proved that modulation of the DA system affects cortical glutamate levels (mainly in the prefrontal lobe) and local glutamate release; glutamate levels can affect DA release in the striatum; and the administration of ketamine can lead to the increase of DA release in the striatum. Low glutamate release or insufficient activation of NMDA receptors on cortical GABA interneurons may lead to hyperactive striatal dopaminergic activity (13, 26–28). Clinically, the use of DA receptor antagonists for ketamine-induced psychosis further provides evidence of the interaction of glutamate and DA (29). Thus, we speculate that the glutamate system can use a common pathway that interacts with the DA system to contribute to the occurrence of psychotic symptoms.

In short, comparing psychotic symptoms induced by the nonmedical use of ketamine and methamphetamine, we have demonstrated that some features of psychotic symptoms in MUD and KUD patients are highly similar to schizophrenia. Our study indicates the significance of identifying and treating psychotic symptoms in MUD and KUD patients, and provides a preliminary basis for clinical identification of the characteristic of psychotic symptoms between schizophrenia and substance use disorder.

## Limitations

This study has some limitations that need to be considered. First of all, we only assessed residual psychotic symptoms but not acute effects of methamphetamine and ketamine. All participants were from voluntary drug rehabilitation centers, and they were not randomly sampled, so the representativeness is limited. Secondly, most patients in the present study were male, so we did not assess gender differences for psychotic symptoms. Thirdly, some drug use variables were different between MUD patients and KUD patients. Compared with MUD patients, KUD patients showed an earlier age of the first use, longer duration of ketamine use, higher quantity of drug use per time, and higher drug craving level. However, this study found more MUD patients experienced psychotic symptoms than KUD patients. The underlying mechanism needs to be further researched. Lastly, some MUD or KUD patients also used other drugs. However, this study excluded patients with other substance use disorders (excluding nicotine).

## Conclusion

In conclusion, this study found that psychotic symptoms are commonly reported by MUD patients and KUD patients from drug rehabilitation centers, and that MUD patients are more likely to suffer from psychotic symptoms (but not general symptoms) than KUD patients. These findings indicate the importance of assessing psychotic symptoms among these two groups of patients. It also provides a new perspective for exploring glutamatergic model and dopaminergic model of psychosis.

## Data Availability Statement

The raw data supporting the conclusions of this article will be made available by the authors, without undue reservation.

## Ethics Statement

The study protocol has been approved by Ethics Committee of the Second Xiangya Hospital, Central South University (No. S163, 2011). The patients/participants provided their written informed consent to participate in this study.

## Author Contributions

YL conceived the study and took the lead in writing the manuscript. YL and MX did the literature review. TL did the statistical analyses. YL, TL, and MX drafted the report. YL, CQ, and QW collected the data. TL, MX, and JT interpreted the data and commented on the manuscript. JT supervised the study. All authors contributed to the article and approved the submitted version.

## Funding

This study was supported by the National Natural Science Foundation of China (Grant No. 81671325 to YL) and the Hunan Provincial Natural Science Foundation of China (Grant No. 2020JJ4794 to YL).

## Conflict of Interest

The authors declare that the research was conducted in the absence of any commercial or financial relationships that could be construed as a potential conflict of interest.

## Publisher's Note

All claims expressed in this article are solely those of the authors and do not necessarily represent those of their affiliated organizations, or those of the publisher, the editors and the reviewers. Any product that may be evaluated in this article, or claim that may be made by its manufacturer, is not guaranteed or endorsed by the publisher.
